# Infrared Linear Dichroism for the Analysis of Molecular Orientation in Polymers and in Polymer Composites

**DOI:** 10.3390/polym14061257

**Published:** 2022-03-21

**Authors:** Liliane Bokobza

**Affiliations:** Independent Researcher, 194-196 Boulevard Bineau, 92200 Neuilly-Sur-Seine, France; liliane.bokobza@wanadoo.fr

**Keywords:** infrared spectroscopy, infrared dichroism, chain orientation, polymers, semi-crystalline polymers, copolymers, polymer blends, polymer composites

## Abstract

The mechanical properties of polymeric materials are strongly affected by molecular orientation occurring under processing conditions. Infrared dichroism is particularly well suited for characterizing polymer chain orientation at a molecular level. The usefulness of this technique has been demonstrated through various applications in homopolymers, semi-crystalline polymers, copolymers, polymer blends, as well as in polymer composites. Determination of molecular orientation can be carried out in the mid- and near-infrared ranges and very small dichroic effects can be detected with the use of a photoelastic modulator. Chain orientation in polymer composites is seen to increase with the filler content in the case of a strong interface between the two phases, making possible a quantification of the degree of bonding between the host polymeric matrix and the incorporated inclusions.

## 1. Introduction

Polymer properties especially mechanical behavior, highly depend on polymer morphology. In addition to the chemical composition, the mechanical properties can be modified by many factors including the orientation of the polymer chains, a phenomenon of great technical and theoretical importance that can occur during stretching or during polymer processing. The knowledge of this phenomenon at a molecular level allows a better understanding of the deformation mechanisms of the material and the establishment of a correlation between the processing conditions and the properties of the fabricated sample. Measurements of orientation have been performed on homopolymers and multicomponent systems [1,2,3,4,5,6,7,8]. Spectroscopic techniques including nuclear magnetic resonance, polarized fluorescence, and polarized vibrational spectroscopies (infrared linear dichroism and polarized Raman) have been used for quantifying molecular orientation [9,10,11,12,13,14,15]. Among these techniques, infrared spectroscopy is one of the most frequently applied since it can be used for the analysis of many oriented polymeric materials and can allow the determination of the orientation of both crystalline and amorphous phases of semicrystalline polymers if the infrared spectrum contains bands associated with vibrational modes specific for each phase. It is also particularly well-suited for the determination of the orientation of the different components of copolymers, polymer blends, or polymer composites. Therefore, this chapter will only concentrate on infrared measurements of uniaxially stretched polymeric systems.

Infrared spectroscopy probes the vibrational states of a molecule, but infrared absorption requires a change in the dipole moment during the vibrational mode considered. The occurrence of almost constant vibrational frequencies associated with the presence of particular chemical functional groups, vibrating independently of the rest of the macromolecule, makes infrared dichroism particularly interesting for an evaluation of the level of orientation of the chemical groups of interest and for the different phases of multicomponent materials [16,17].

The careful analysis of segmental orientation in polymeric systems has brought indispensable information on the intrinsic conformational properties of the macromolecular chains. After recalling the basic principles of infrared linear dichroism, we present some applications in homopolymers, semi-crystalline polymers, copolymers, polymer blends, and in polymer composites.

## 2. Definition of Orientation and Orientation Functions

**Figure 1:**
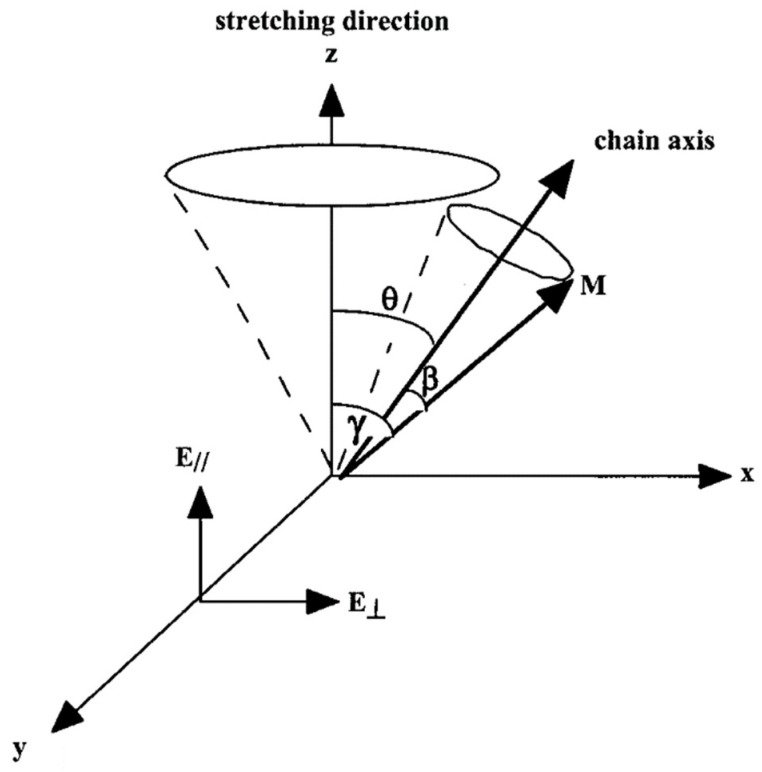
Definition of the axis system and angles to describe polymer orientation. Source: Reprinted with permission from Bokobza, et al., 2000.

The absorption of infrared radiation results from the interaction between the electric field vector of the incident light, E and the electric dipole-transition moment, M, of a given vibrational mode. For linearly polarized radiation, the absorbance A of a band associated with a particular vibrational mode is proportional to the square of the scalar product of M and E, which in turn is proportional to the square cosine of the angle γ between the dipole-transition moment and a reference axis (z), which can be taken as the stretching direction of a polymer film (Figure 1):

(1) $$\;A\;\propto \;(M.\;E){}^{2}\;\propto \;(\mathrm{ME}){}^{2}\;\cos{}^{2}\;$$

The absorbance is maximum when γ = 0 and is zero when γ = 90°.

For a uniaxially oriented sample, with the electric vector respectively parallel and perpendicular to the symmetry axis (which coincides with the direction of stretch):

(2) $$\;A{}_{//}\;\propto \;M^{2}\;E^{2}\;<\cos{}^{2}\;\gamma >\;$$

(3) $$A{}_{⊥}\;\propto \;M^{2}\;E^{2}\;1/2\;<\sin{}^{2}\gamma >\;$$

<cos^2^ γ> can be obtained by combining Equations (2) and (3):

(4) $$<\cos{}^{2}\;\gamma >=\frac{\;A{}_{//}}{A{}_{//}+2A{}_{⊥}\;}$$

A_//_ and A_⊥_ are the absorbances of the investigated band measured with light polarized parallel and perpendicular to the z-axis. One can then compute the dichroic ratio R (R = A_//_/A_⊥_) or the dichroic difference ΔA (ΔA = A_//_ − A_⊥_); these two parameters are commonly measured to characterize molecular orientation in drawn polymers.

The orientation of the transition moment vector itself with respect to the direction of stretch is expressed in terms of the second Legendre polynomial 〈P_2_ (cos γ)〉 related to the dichroic ratio by the expression:

(5) $${<P}_{2\;}(\cos\gamma >=\frac{1}{2}{\;(3\cos}^{2}\gamma \;-\;1)=\frac{R\;-\;1}{R+2}$$

The quantity (R − 1)/(R + 2) is the dichroic function and can be accessed experimentally. The dichroic function is equal to 0 if R = 1, no anisotropy is detected. This is the case if the sample is isotropic or if cos^2^ γ = 1/3 (γ~55°). It has also to be mentioned that the derived orientation is an average over all the identical chemical groups in a given polymer chain, and over all the identical polymer chains present in the sample.

The average orientation of several transition dipoles associated with different absorption bands which precludes any assumption regarding the local chain axis whose orientation—which is the quantity of interest—is given in infrared spectroscopy, by the second moment of the orientation function, <P_2_ (cos θ)> = [3 <cos^2^ θ> − 1]/2 (θ being the angle between the chain axis and the uniaxially stretched direction).

If the direction of the dipole transition moment makes an angle β with the chain axis (Figure 1), according to the Legendre addition theorem, the average second moment of the orientation function, <P_2_ (cos θ)> is related to <P_2_ (cos γ)> by the simple relationship:

(6) $$<P{}_{2}\;(\cos\;\gamma )>=<P{}_{2}\;(\cos\;\beta )><P{}_{2}\;(\cos\;\theta )>$$

<P_2_ (cosθ)> is thus related to the dichroic ratio R by the following expression:

(7) $${<P}_{2}\;(\cos\;\theta )>=\frac{2}{\left(3\;\cos^{2}\beta -1\right)}\frac{\left(R-1\right)}{\left(R\;+2\right)}$$

Determination of <P_2_ (cos θ)> requires the knowledge of the angle β between the transition moment vector of the vibrational mode considered and the chain axis. β can theoretically be obtained from considerations arising from group theory provided that the absorbing functional groups have some symmetry elements. Alternatively, if the second moment of the orientation function, <P_2_ (cos θ)>, is known by using a well-defined absorption band, the angle β related to any other infrared absorption band can be determined.

Measurements are usually performed with the use of a linear polarizer, but they can be significantly improved by the introduction of polarization modulation technique where a photoelastic modulator, placed in the beam of light, alternates the polarization state of the incident radiation between directions parallel and perpendicular to the stretching axis, thus allowing a direct measurement of the dichroic difference ΔA [18,19]. This technique allows the detection of very low dichroic effects observed at small extension ratios or in polymers that display low levels of orientation due to their high chain flexibility like poly(dimethylsiloxane) (PDMS) [20].

Infrared dichroism measurements by the conventional transmission method, require the use of absorption bands whose absorbance should be roughly lower than 0.7 to allow application of the Beer–Lambert law. This implies the use of thin polymer films of thickness often less than 100 μm. In thick films, fundamental vibrational modes often give rise to strong absorption bands located in the classical mi-infrared (mid-IR). In that case, overtones and combinations, much weaker than the fundamental vibrations, located in the mid- or near-infrared (NIR) range, can be examined. In the NIR region, between 4000 and 12,500 cm^−1^, bands are found associated with overtones and combinations of fundamental vibrations of hydrogen-containing groups such as C–H, N–H, and O–H [21,22,23,24].

**Figure 2:**
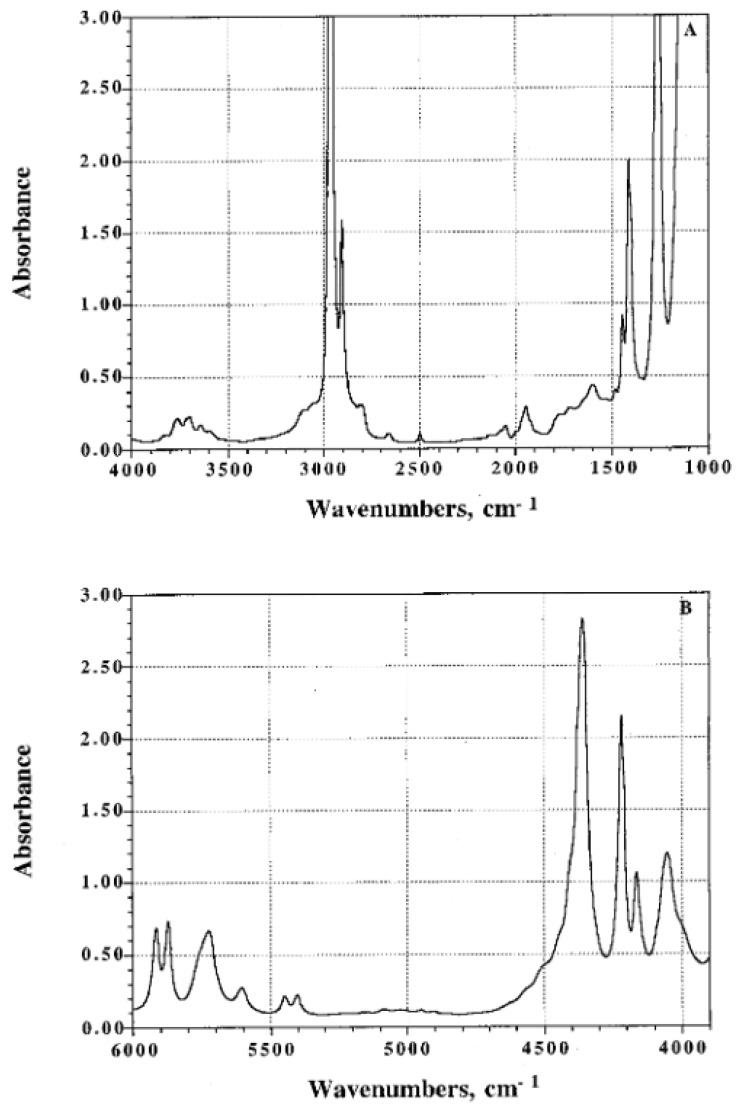
Infrared spectra of poly(dimethylsiloxane) (PDMS) films: mid-infrared of a 125 μm thick film (**A**); near-infrared spectrum of a 2 mm thick film (**B**). Source: Reprinted with permission from Ref. [25].

**Figure 3:**
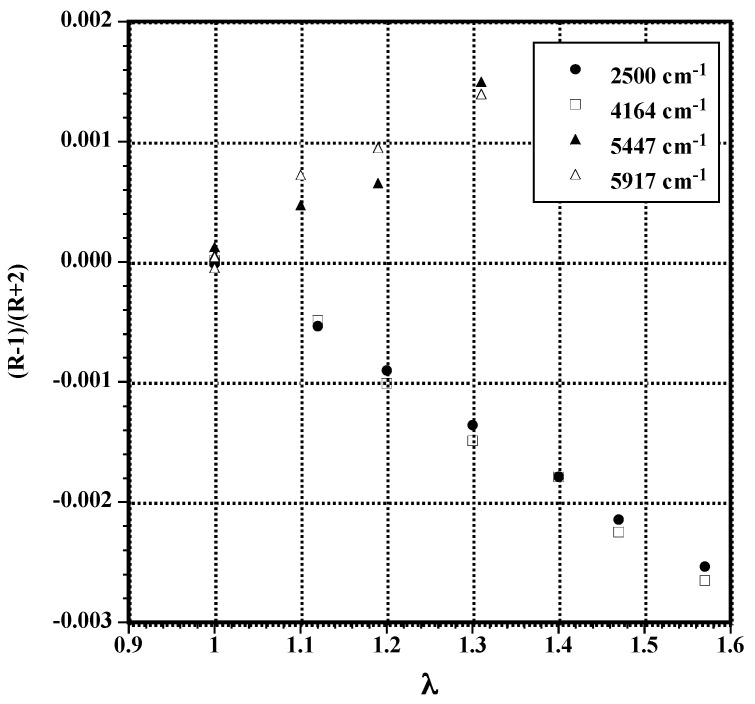
Dichroic functions vs. uniaxial draw ration of different infrared absorption bands of PDMS films located in the mid- and NIR ranges.

As a typical example, mid- and NIR infrared spectra of poly(dimethylsiloxane) (PDMS) films are displayed in Figure 2. As seen in Figure 2A, most of the bands associated with the fundamental modes are very strong even at a thickness of 125 μm. Dichroic measurements can be performed on the band located at 2500 cm^−1^ ascribed to the overtone of the symmetrical bending vibration δ_s_ (CH_3_) located at 1260 cm^−1^ [25]. On account of its weak extinction coefficient, the dichroic behavior of the band at 2500 cm^−1^ can also be investigated in samples up to 2 mm thick. Otherwise, several absorption bands can be used in the NIR range for orientational measurements (Figure 2B). In Figure 3 the dichroic functions (R − 1)/(R + 2) of several infrared bands are plotted as a function of the uniaxial draw ratio, λ, defined as the ratio of the final length of the sample along the direction of stretch to that of the initial length before deformation. The dichroic function of the bands at 2500 and 4164 cm^−1^ decreases with an increase in uniaxial deformation contrary to that of the bands located at 5447 and 5917 cm^−1^ which exhibits opposite behavior. The dichroic ratio of the bands at 2500 and 4164 cm^−1^ is <1 thus proving that the corresponding transition moment is most likely perpendicular to the directional vector while that associated with the bands at 5447 and 5917 cm^−1^ is parallel. All the investigated bands are assigned to vibrational states of the methyl group and obviously belong to two different symmetry species (A_1_ and E of the C_3v_ point group of CH_3_).

Chain orientation of thicker polyethylene sheets under uniaxial orientation under uniaxial deformation was also characterized by near infrared spectroscopy by Mizushima et al. [26]. The authors used the bands at 1728 and 1754 nm assigned to the first overtone of the asymmetric and symmetric stretching vibrations of the CH_2_ groups.

## 3. Orientation of Homopolymers, Semi-Crystalline Polymers, Copolymers, Polymer Blends

**Figure 4:**
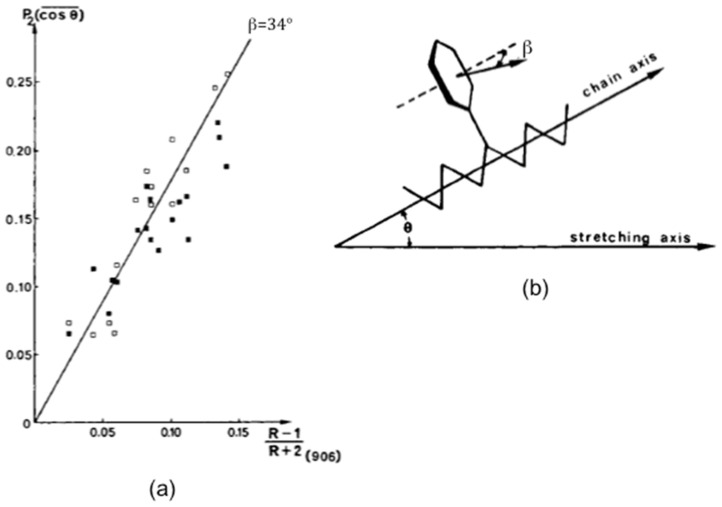
Example of determination of the angle β between the transition moment vector associated with the band at 906 cm^−1^ and the chain axis in atactic polystyrene from infrared measurements carried out at 100 °C for the bands located at 1028 cm^−1^ (∎) and 2850 cm^−1^ (⧠) (**a**) and definition of the angles in a segment of polystyrene in trans conformation (**b**). Source: Reprinted with permission from Ref. [27].

Fourier transform infrared (FTIR) spectroscopy has been applied to evaluate the orientation of atactic polystyrene films uniaxially drawn at 100 °C, in the glass-transition region and at 110 °C, above the glass transition [27]. Measurements were performed on samples quickly cooled under stress to room temperature after drawing at the adequate temperature, in order to freeze the state of chain orientation. The dichroic ratio R as a function of the draw ratio was determined for the bands located at 2850, 1028, 906, and 540 cm^−1^. While the 540 cm^−1^, associated with an out-of-plane vibration of the aromatic ring, is connected with a structure with at least four aliphatic backbone chain bonds in trans conformation, the other investigated bands are conformationally insensitive. The second moment of the orientation function <P_2_ (cos θ)> was determined from the dichroic ratio of the bands located at 1028 and 2850 cm^−1^ respectively ascribed to the in-plane CH bending normal mode of the aromatic ring and to the CH_2_ symmetrical stretching with a dipole moment vector perpendicular to the chain axis. An angle β of 90° is taken into account for the band at 1028 cm^−1^ but a value of 70° is chosen for the band at 2850 cm^−1^ as that found in polyethylene. The out-of-plane mode of the ring giving rise to the absorption at 906 cm^−1^, presents a dipole moment vector perpendicular to the plane of the benzene ring that makes an angle β with the chain axis as seen in Figure 4b. This angle can be determined by plotting <P_2_ (cos θ)> calculated from the measurements carried out on the bands at 1028 and 2850 cm^−1^ against the dichroic function of the absorption at 906 cm^−1^ according to Equation (7). It was also shown that the orientation process leads to an increase in the amount of trans conformational segments and increasing the temperature decreases the overall orientation of the chains.

### 3.1. Semi-Crystalline Polymers

The strain-induced crystallization phenomenon in cross-linked natural rubber (NR), discovered in 1925 but widely discussed in the literature [28,29,30,31], explains the high values of the stress at rupture and the maximum extensibility. This important characteristic attributed to the uniform microstructure of NR (cis configuration of the macromolecular chains), may be regarded as a “self-reinforcing effect”. The formation of crystallites which may be considered as additional cross-links in the polymer network, able to align along the drawing axis, explain the exceptional mechanical properties of NR. The strain-induced crystallization of stretched NR is evidenced by the shift of the C–H out-of-plane absorption band [32,33] and the respective contributions to this band allow the determination of the polymer chain orientation in the amorphous and crystalline phases.

The orientation of subunits can be determined if some absorption bands are specific of a given conformation or configuration.

Polyethylene terephthalate, PET, is an important thermoplastic polymer, commonly used for the production of fibers, food, and liquid packaging. It is a semi-crystalline polymer and may contain amorphous and crystalline phases. One peculiarity of PET is the existence of gauche and trans conformers formed by rotation around the bonds of the ethylene glycol group. Cis and trans conformers can also be formed by rotation of the carbonyl groups around the aromatic group. The conformational changes induced by drawing or thermal annealing can be followed by infrared spectroscopy [34,35,36,37].

Cole et al. [35] used specular reflection FT-IR spectroscopy to study thick films of amorphous PET submitted to uniaxial stretching or thermal annealing. PET in the amorphous state is primarily constituted of gauche conformers that convert into trans conformers upon uniaxially stretching the film at 80 °C. These conformational changes are of great practical interest since they determine the degree of crystallinity and orientation that strongly impact the physicochemical behavior of the material. The orientation of the gauche and trans conformers was evaluated through the determination of the dichroic function (R − 1)/(R + 2) of the bands located at 1370 cm^−1^ and 1340 cm^−1^ respectively assigned to the CH_2_ wagging mode of the gauche and trans segments. While gauche conformers do not exhibit detectable orientation up to an extension ratio of 5, the trans conformers orient from the start of drawing and reach a high level of orientation. Interestingly, the carbonyl band of the amorphous sample stretched at 80 °C does not display any change of its wavenumber located at 1723 cm^−1^ up to an extension ratio of 5 while it shifts to 1719 cm^−1^ and does not change upon drawing in the case of the thermally crystallized sample obtained by annealing the amorphous material at 200 °C for 15 min. The authors suggested a possible cis to trans conversion.

Molecular orientation of the trans and gauche conformers for a PET film stretched at 85 °C was also determined by Duchesne et al. [36] by the analysis of the dichroic behavior of the bands located at 1340 and 1370 cm^−1^ but by using the polarization modulation technique that allows the measurement of small dichroic effects with a high sensitivity. The second moment of the orientation function, <P_2_ (cos θ)> is calculated from the observed dichroic differences, ΔA (ΔA = A_//_ − A_⊥_), according to Equation (8), obtained by rewriting Equation (7) and making appear the structural absorbance A = (A_//_ + 2A_⊥_)/3 that decreases as A_0_/$\sqrt{\lambda \;\;}$ (A_0_ being the absorbance under isotropic conditions) on account of reduction in film thickness during uniaxial deformation.

(8) $${\;<P}_{2}(\cos\;\theta )>=\frac{2}{\left(3\cos^{2}\beta -1\right)}\frac{\left(R-1\right)}{\left(R+2\right)}=\frac{2}{\left(3\cos^{2}\beta -1\right)}\;\frac{A_{//}-A_{⊥}}{A_{//}+2A_{⊥}}=\frac{2}{\left(3\cos^{2}\beta -1\right)}\;\frac{\Delta A}{3A_{0}}\sqrt{\lambda }$$

**Figure 5:**
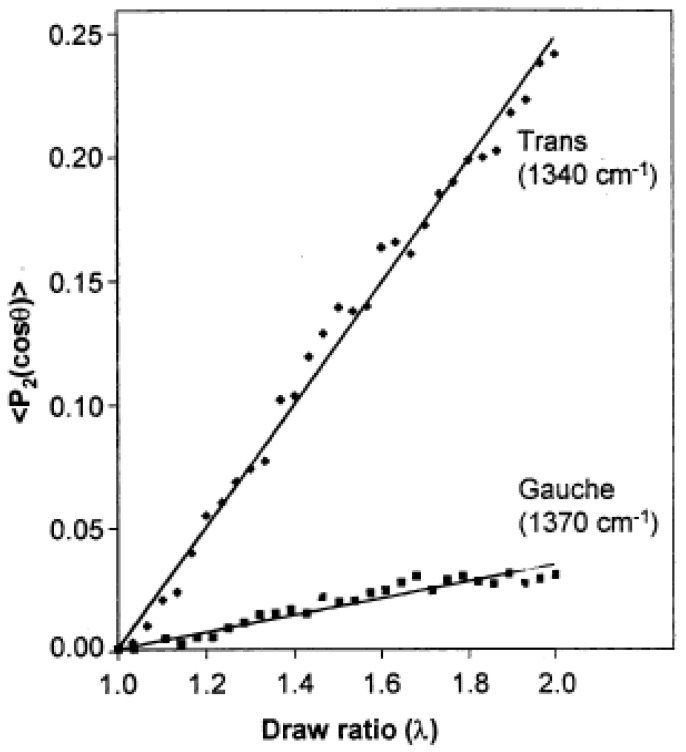
Second moment of the orientation function of the trans and gauche conformers of PET stretched uniaxially at 85 °C at a constant draw rate of 10 cm/min. Source: Reprinted with permission from Ref. [36].

Figure 5 shows the second moment of the orientation function of trans and gauche conformers as a function of the extension ratio, for samples stretched up to λ = 2 at 85 °C. In both cases, the increase is linear with the draw ratio but the level of orientation of the gauche isomers is much smaller than that of the trans conformers but can nevertheless be evaluated by the polarization modulation infrared linear dichroism.

### 3.2. Copolymers

Information on the orientation of specific parts of a polymer chain can be obtained by deuterium labeling to produce block copolymers, for example, with hydrogenated and deuterated blocks. Infrared spectroscopy is able to characterize the orientation of each species owing to the wavenumber shift on deuteration due to the mass dependence of the vibrational frequency.

By using deuterium labelled block copolymers—a diblock and a triblock copolymers [poly(d_8_-styrene-b styrene) and poly(styrene b-d_8_ styrene b styrene)—Tassin et al. [38] were able to extract the orientation of the middle and the chain ends. The dichroic ratios of the bands at 2273 and 2195 cm^−1^, respectively assigned to a stretching vibration of the aromatic C–D group and to the asymmetric stretching mode of the CD_2_ groups, were measured for samples uniaxially stretched, at a constant strain rate, at different draw ratios and at various temperatures above the glass transition temperature. It was shown that the central block has a higher orientation than the end portions and the difference between the two levels of orientation increases with temperature.

The molecular orientation of poly(styrene-block-butadiene-block-styrene) triblock copolymer (SBS) with uniaxial strain has been shown to depend on the morphology of the undrawn films obtained by changing the casting solvent [39]. Polystyrene (PS)–polybutadiene (PB) alternating lamellar, PB-cylindrical and PS–PB bicontinuous microdomain structures were obtained by using toluene, methyl ethyl ketone (MEK), and heptane, respectively. The dichroic functions, determined from the dichroic ratios of the bands at 1493 and 966 cm^−1^ for PS and PB, respectively, display negative values with strain indicating that the corresponding transitions moments are perpendicular to the local chain axis. For all strains and all morphologies, the absolute values of the dichroic functions of PS are smaller than those of PB. For each specimen, the strain dependence of the orientation of the PS and PB chains is discussed in relation to the stress–strain behavior.

Orientation in deformed elastomeric networks has been the subject of considerable attention because the new experimental developments in the evaluation of segmental orientation yielding precise measurements of the dichroic effects, have allowed the test of theoretical predictions [40]. The second moment of the orientation function is related to the extension ratio, λ, by a series expansion [41] whose first term is:

<P_2_ (cos θ)> = D_0_ (λ^2^ − λ^−1^) (9)

The prefactor D_0_ (called the “configurational factor”), which takes into account the structural features of the network chains, is inversely proportional to the average molecular weight between crosslinks, M_c_ [42]. The second term, (λ^2^ − λ^−1^), that relates the orientation to the macroscopic deformation, is called the strain function. Equation (9) is related to a network where chains are assumed to deform affinely with the macroscopic deformation.

**Figure 6:**
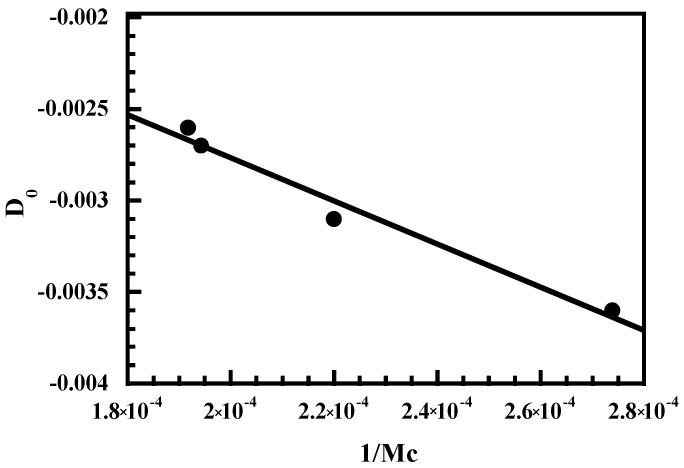
Configurational factor of styrene–butadiene copolymers against the reciprocal molecular weight between the crosslinks, M_c_.

Elastomeric networks with random styrene–butadiene copolymers exhibiting a similar styrene content but varying butadiene microstructure (cis, trans, vinyl configurations) were investigated [43,44]. The dichroic behavior of the bands located at 1640, 1493, and 4477 cm^−1^ respectively ascribed to the C=C stretching vibration of the vinyl unit, a benzene ring vibration, and to a combination of a stretching and a bending mode of the vinyl group was investigated. D_0_ is determined from the slopes of the curves representing the orientation of the transition moment vector, <P_2_ (cos γ)> = (R − 1)/(R + 2), versus the strain function, (λ^2^ − λ^−1^). The three bands exhibit a negative orientation (negative D_0_ or <P_2_ (cos γ)>) with strain, thus showing that their corresponding transition moment is perpendicular to the local chain axis. As shown in Figure 6, a linear relation is observed between the configurational factor, D_0_ and 1/M_c_ which is in good agreement with the theory stating that infrared dichroism is controlled by the molecular weight between crosslinks determined from stress–strain curves or equilibrium swelling experiments.

### 3.3. Polymer Blends

Determination of the orientation of the two components of polymer blends can bring a better understanding of the mechanisms of deformation and relaxations of these components [45].

**Figure 7:**
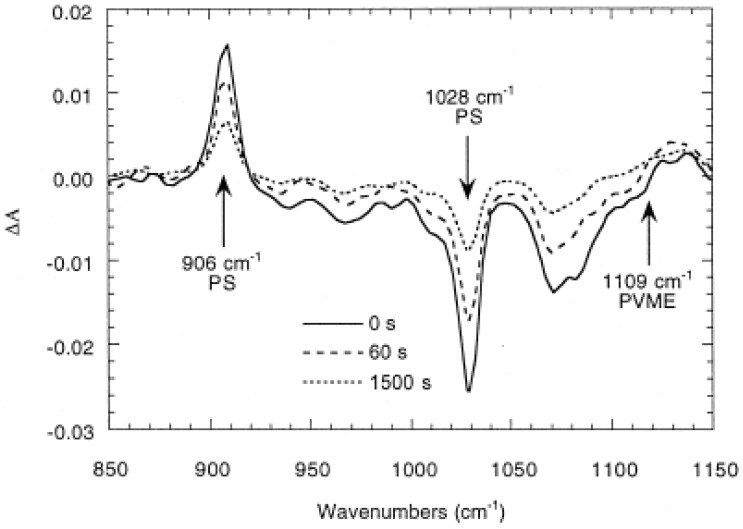
Dichroic difference spectra during the relaxation period of a PS–PVME blend containing 90% PS stretched to a draw ratio of 2 at T_g_ + 8. Source: Reprinted with permission from Ref. [46].

The high sensitivity of the polarization modulation technique in measuring infrared linear dichroism allows a detailed analysis of the dynamics of orientation of polymer blends. Real-time dichroic difference spectra were recorded during the relaxation period of a stretched films of polystyrene (PS) and poly(vinyl methyl ether) (PVME) blend [46]. The small dichroic effect observed for the shoulder at 1109 cm^−1^ due to the C–O–C asymmetric stretching mode of the methoxy side chain of PVME indicates that the chains of PVME remain practically unoriented. On the other hand, the dichroic difference of the bands decreases with time due to the relaxation of the chains to their isotropic state (Figure 7). For each PS–PVME blend, the second moment of the orientation function of PS increases linearly with the draw ratio and with the PVME content.

**Figure 8:**
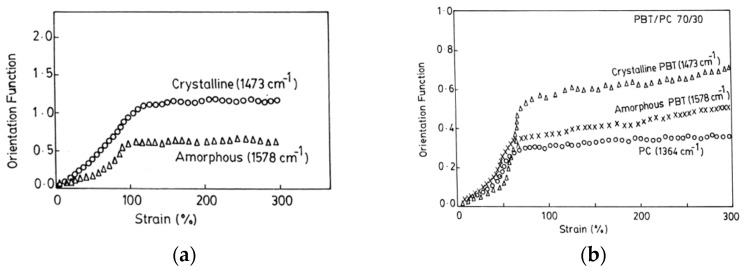
Strain dependence of the orientation during uniaxial deformation at 80 °C of the crystalline and amorphous phases in the pure PBT (**a**) and in the 70/30PBT/PC blend in addition to that of the PC chains (**b**). Source: Reprinted with permission from Ref. [47].

Rheo-optical Fourier transform infrared spectroscopy, which combines mechanical measurements and linear infrared dichroism, has been used to analyze the orientation and relaxation in uniaxially drawn films of semicrystalline partial miscible blends of poly(butylene terephthalate) (PBT) with polycarbonate (PC) containing 10, 30, and 50 wt% PC [47]. The second moment of the orientation function of pure PBT determined from the dichroic behavior of the bands at 1473 cm^−1^ (CH_2_ deformation in crystalline region) and 1578 cm^−1^ (symmetric stretching vibration of the phenylene ring in the amorphous region) is much larger for the crystalline segments compared to the amorphous ones (Figure 8a). PBT in the blend exhibits a lower degree of crystalline orientation (Figure 8b) compared to the pure polymer thus showing that the incorporation of PC hinders the crystallization of PBT in the blends. For all the blend compositions, the orientation of the amorphous PBT component is lower than that of the pure amorphous PBT. On the other hand, the overall lower orientation of the PC chains in the blends, determined from the band at 1364 cm^−1^ assigned to the in phase symmetrical bending vibration of the two methyl groups, is explained by the stretching temperature which is much lower than the T_g_ of PC (145 °C).

## 4. Orientation of Polymer Composites

Inorganic particles such as carbon black or silica have been widely used to prepare polymer composites with greatly improved properties compared to the pristine polymer [48,49,50,51,52,53]. The extent of property improvement depends on the morphology of the particles, their state of dispersion in the host medium, and essentially on the polymer–filler interfacial interactions. Nanoscale fillers with isotropic or anisotropic sheet-like or needle-like morphologies, bring much improved properties when introduced in the host matrix at a very low content on account of their nanoscale dimensions that create a very large polymer–particle interfacial region. This interfacial region has a major importance in nanocomposite properties and analyzing its characteristics and its role on the macroscopic properties of the material allows the design of composites with specific applications.

Different filler morphologies such as nanospheres, nanotubes, or nanoplatelets, have been used for polymer reinforcement. Anisotropic fillers of one or two-dimensional nature are able to orientate during processing or mechanical stretching which has a strong effect on the reinforcement of the resulting material in the direction of alignment. Infrared spectroscopy is a powerful tool for the analysis of polymer composites. It can be used for the identification of chemical groups on the filler surface, the grafting of specific molecules for modifying the surface reactivity, and the interacting species at the polymer–filler interface. Orientational measurements of uniaxially stretched composites by infrared linear dichroism, nicely complement the mechanical data because, as shown below, polymer chain orientation is sensitive to the interfacial bonding between the two components as well as to the filler morphology.

**Figure 9:**
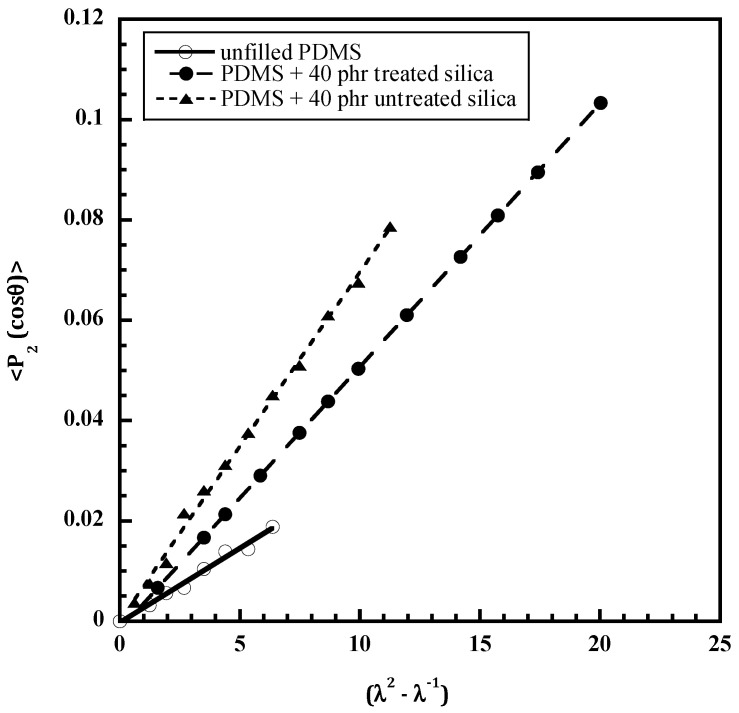
Polymer chain orientation in unfilled and filled poly(dimethylsiloxane) with 40 phr of untreated and treated silica. “phr” = parts of filler per hundred parts of rubber.

In silica-filled systems, hydrogen bonding can take place between the silanols present on the silica surfaces and the oxygen-containing functional groups of polymers such as PDMS, poly(methyl methacrylate), poly(vinyl acetate) or epoxy resins [54,55,56,57,58,59]. Polymer–filler interactions can be tuned by treating silica particles with a processing aid in order to deactivate part of the silanols. The addition of silica in PDMS leads to increases in the modulus, tensile strength, and elongation at break. Deactivating part of the silanols decreases the number of reactive groups at the polymer–filler interface resulting in less improvement of the mechanical properties than with the use of untreated particles. As seen in Figure 9 the second moment of the orientation function of the PDMS chains, determined from the dichroic behavior of the band at 2500 cm^−1^, increases linearly with the strain function, (λ^2^ − λ^−1^) for each sample while at the same extension ratio, λ, it increases with a more pronounced effect in the case of untreated particles. The increase in chain orientation upon addition of filler is attributed to polymer–filler interactions by hydrogen bonding between the silanols on the silica surface and the oxygen atoms of PDMS. These interfacial interactions that act as additional cross-links in the network structure increase with the filler content or with the interface area of the polymer–filler system. In the case of treated silica, the number of additional cross-links has been found to be 0.13 nm^−2^ (corresponding to a bonding around every 6 nm^2^ of silica surface) and 0.3 nm^−2^ for the treated silica [60]. Therefore, infrared linear dichroism applied to uniaxially stretched filled elastomers, appears to be an interesting technique for evaluation of the number of polymer–filler attachments.

In the case of poor compatibility between the organic and inorganic phases, as in silica-filled hydrocarbon rubbers, silane coupling agents such as bis(3 triethoxysilylpropyl)tetrasulfide (TESPT), commonly abbreviated “Si69”, are used to improve the adhesion between the elastomer chains and the filler surface. These coupling agents are generally bifunctional molecules able to react with the polymer matrix and the particle surface. However, on account of the tetrasulfane function of the TESPT which can react with the polymer during the curing process, it is not clear if TESPT leads to an increase of in the cross-linking density rather than to an interfacial coupling. It has been shown that in the absence of the coupling agent, the orientational behavior of polymer chains in silica-filled styrene–butadiene rubbers is similar to that of the unfilled network while it increases with the filler fraction in the presence of Si69 thus reflecting the quality of the polymer–filler interface [61,62].

Xu et al. [63] analyzed the orientation by infrared dichroism of stretched films of neat polyaniline (PANI) and its nanocomposites with reduced graphene oxide (rGO) or multi-walled carbon nanotubes (MWCNTs) in order to obtain an insight into the effect of molecular orientation on the thermoelectric performance of polymeric materials. The dichroic ratios of the bands located at 1594, 1313, and 1167 cm^−1^ increase upon stretching contrary to that of the band at 833 cm^−1^ which decreases with uniaxial deformation. The addition of MWCNTs or rGO reduces the orientation of PANI and the hindrance effect increases with the filler content and is higher with rGO than with MWCNTs. No mention is made by the authors on the interfacial interactions between polymer and nanoparticles.

Incorporation of a fibrous clay such as sepiolite in natural rubber (NR) has been shown to strongly affect the mechanical and orientational properties of the final material [64,65]. Sepiolite is a hydrous magnesium silicate with a crystal structure formed by two sheets of tetrahedral silia units to a central sheet of magnesium atoms. The sepiolite (Pangel B20) used as a filler for NR is an organophilic fibrous clay, obtained from the pristine sepiolite by physico-chemical purification, micronization, and chemical modification processes developed and patented by Tolsa. In particular, the surface treatment of sepiolite by surfactants makes the nanofibers of sepiolite more compatible with low polarity polymers.

**Figure 10:**
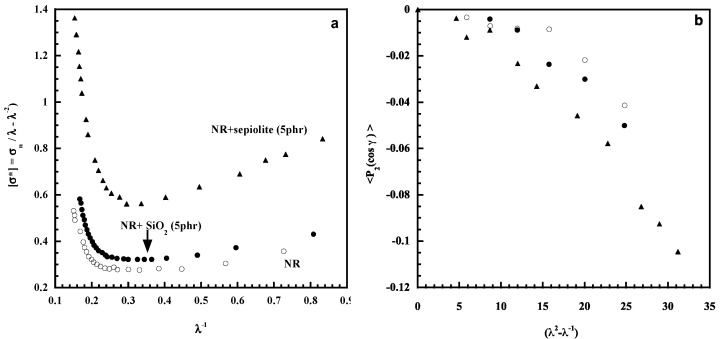
Reduced stress as a function of reciprocal elongation for pure NR and for composites (**a**); orientational measurements for the same materials (**b**). Source: Reprinted with permission from Refs. [64,65].

Mechanical data of pure NR and for NR filled with two different fillers—silica particles generated in situ by the sol-gel process and sepiolite—are shown in Figure 10a. They are treated in the Mooney–Rivlin representation by calculating the reduced stress, [σ*], defined by the quantity: [σ*] = σ_n_/(λ – λ^−2^), where σ_n_ is the nominal stress and λ the extension ratio [66,67]. This way of plotting the mechanical data gives a better visualization of specific features of the stress–strain curve in particular the upturn in the modulus observed at high deformations and ascribed to the strain-induced crystallization of NR. In the presence of filler particles, the upturn starts at a lower deformation than in the unfilled sample. Moreover, the upturn occurs at a lower strain with sepiolite than with silica. Strain amplification effects due to the inclusion of non-deformable particles cause overstraining of the polymer chains making them become more oriented and then able to crystallize at a lower applied extension ratio.

The orientation of the transition moment associated with the band located at 4291 cm^−1^ that exhibits a perpendicular dichroism (R < 1) is displayed in Figure 10b for pure NR and the two composites. It is clearly shown that, at a given deformation, a higher level of orientation is obtained for the composite filled with nanofibers of sepiolite. The orientation of the sepiolite particles in the stretching direction is expected to induce an orientation of the polymer chains along the fiber axis. Additionally, interfacial bonding between the sepiolite and polymer chains was evidenced through the equilibrium swelling behavior of the composite that exhibits a restricted solvent swelling compared to the unfilled sample [64,65]. An excellent compatibility between natural rubber and the organophilic sepiolite was also observed by Satyanarayana et al. [68]

**Figure 11:**
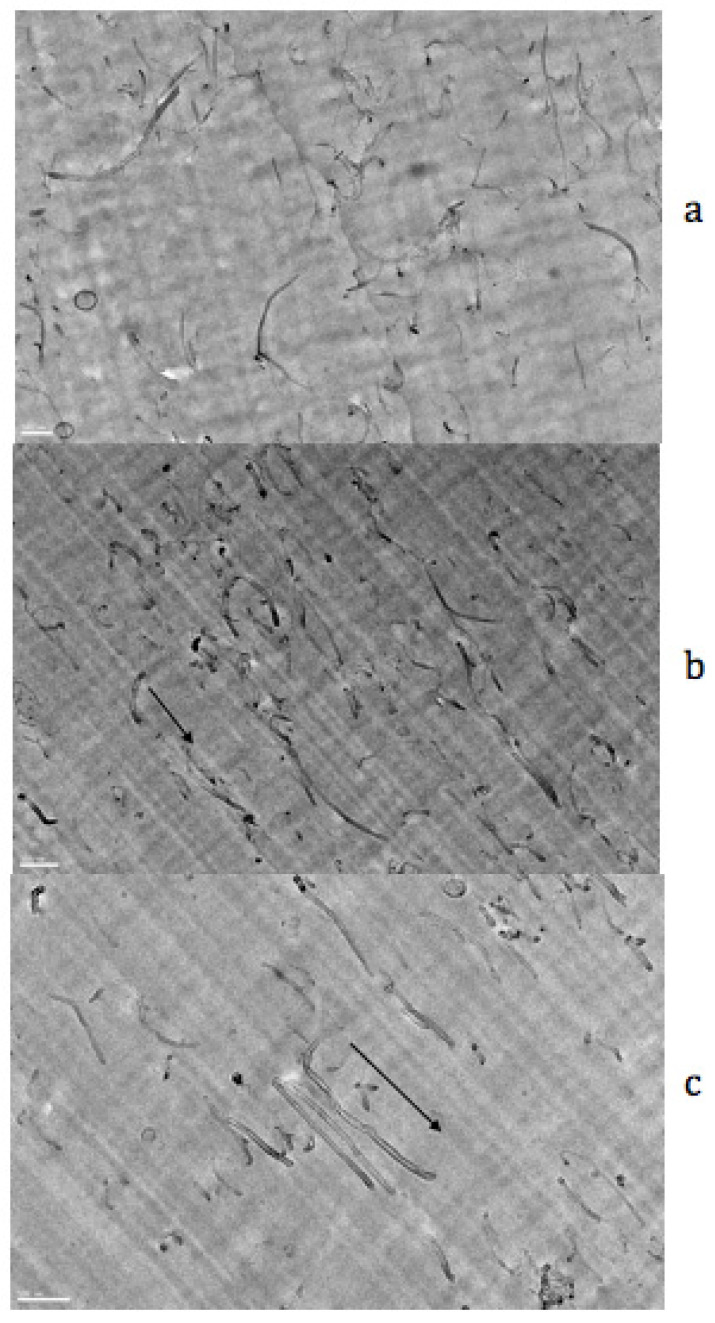
TEM images of thin sections of PS/1 wt% MWCNTs composite in the unstretched (**a**) and stretched at a draw ratio of 4 (**b**,**c**) states. All the scale bars are 100 nm and the arrows indicate the stretching direction. Source: Reprinted with permission from Ref. [73] for Figure 11c.

Other studies have used sepiolite fibers as filler in various polymeric matrices on account of their intrinsically anisotropic character and their orienting capability upon uniaxial drawing [69,70,71,72]. In the work of Hirayama et al. [72] performed on polyvinylidene fluoride/fluorinated phosphonate-modified sepiolite composites, transmission electron microscopy imaging of uniaxially drawn films reveals a high degree of orientation of the needle-shaped nanoparticles along the stretching direction. The mechanical properties of the composites are seen to be significantly enhanced as a result of the uniaxial orientation of the dispersed nanofiller. The high anisotropy of carbon nanotubes also explains their ability to align along the direction of strain as seen in the uniaxially stretched composites of polystyrene (PS) filled with 1 wt% of multiwall carbon nanotubes (MWCNTs) (Figure 11). The unfilled and filled PS films were drawn in the melt state of the polymer at a draw ratio of 4. Then the temperature was rapidly decreased below the glass transition temperature of PS to freeze the state of orientation [73]. Infrared dichroic measurements carried out on PS absorption bands located at 2848, 1028, and 906 cm^−1^ are quite similar in the pure polymer and in the composite. This reflects the poor interfacial interactions between the PS and carbon nanotubes. Functionalization of the filler surface can be used in this case to enhance the interfacial adhesion and to improve the particle dispersion [74].

It has to be mentioned that transmission measurements of polymers filled with carbon-based materials can only be carried out at very low filler content. Transmission measurements are difficult for highly loaded carbon-filled polymers due to the absorption and scattering of infrared radiation by the carbon species [75]. Attenuated total reflectance spectroscopy (ATR) has been shown to offer an alternative to transmission measurements essentially for highly carbon-filled rubbers used in industrial applications [76,77] or for measurement of molecular orientation [78]. Although not considered here, polarized Raman spectroscopy is a powerful tool for the study of molecular orientation distributions of polymers [15,79] This technique that measures inelastic photon scattering, also yield information on the vibrational states of molecules. Its advantage over infrared spectroscopy is to allow the analysis of thick polymer films and black samples. In recent years, this technique has been subject to considerable interest after the discovery of carbon nanotubes because carbon-based fillers display strong resonance-enhanced Raman scattering effects that give rise to strong well-defined bands even if used in very small amounts [52,80]. The strong intensity enhancement makes possible the evaluation of molecular orientation of both polymer chains and carbon species [81].

It is of interest to mention that theoretical and simulation work has been carried out to understand the effect of deformation on the orientation and alignment of polymer chains and anisotropic nanoparticles and the dynamic properties of polymers at the polymer–filler interface [82,83]. Zheng et al. [82] used molecular dynamics simulation to study polymers filled with graphene and carbon nanotubes and studied the effect of a dynamic periodic shear deformation on the orientation and alignment of the anisotropic particles and on the resulting mechanical response by changing the shear amplitude, frequency, interfacial interaction, and volume fraction of filler. Increase in the interaction strength between the two phases and the orientation of the polymer chains are shown to have a significant effect on the resulting mechanical performance. The system filled with CNTS exhibits better mechanical reinforcement than that filled with graphene which has been attributed to the fact that CNTs induce the alignment of polymer chains more along the deformation direction. In the work of Azimi et al. [83], molecular dynamics simulations are conducted on graphene (G) and graphene oxide (GO)-based nanocomposites. One apolar (polypropylene) and one polar (polyvinyl alcohol) polymer matrices were chosen in order to cover a wide range of polymer–filler interactions. Polymer chain orientation was found more pronounced for the G-based nanocomposites than for the GO ones and it is assumed to be attributed to the roughness of the GO surface. It is well established that GO exhibits structural defects induced by the oxidation process resulting in the presence of oxygen-containing functional groups and sp^3^ carbon atoms.

## 5. Conclusions

This paper describes several applications of infrared linear dichroism for characterizing molecular orientation in polymers and in polymer composites. Besides conventional measurements, near-infrared spectroscopy (NIR) or the polarization modulation technique have broadened the use of infrared spectroscopy for the analysis of chain orientation. NIR that probes overtones and combinations of fundamental modes, offers a useful way to study thick polymer films. The polarization modulation technique that uses a photoelastic modulator can be applied in the mid- and near-infrared range to detect very low dichroic effects with a high sensitivity at very small film deformations. The ability to detect molecular orientation in a material is quite important for a better understanding of the mechanisms involved in polymer deformation in order to correlate the processing conditions with the properties of the fabricated sample.

The effect of fillers on the orientation of the polymeric material has been investigated. Orientational measurements of polymer composites can be used to evaluate the degree of bonding between the polymer and the filler. In strongly interacting systems as in the case of silica-filled poly(dimethylsiloxane) networks, chain orientation taking place on uniaxially stretched films yields access to the total network density arising from chemical junctions and from polymer–filler interactions acting as additional cross-links. No change in the orientational behavior of polymer chains is observed in the absence of polymer–filler interactions as in the case of styrene–butadiene elastomeric networks filled with silica particles, unless using a coupling agent is able to bring some coupling between the two phases. Fillers with a high aspect ratio such as clay nanofibers, graphene, or carbon nanotubes, are widely used because they impart to the composites excellent mechanical properties on account of their ability to align in the direction of the strain. The results regarding the effect of these anisotropic particles on the molecular orientation of polymer chains are not clear because they more likely depend on interfacial bonding and on the filler agglomeration. Concerning the black fillers, their own orientation within the host matrix as well as that of macromolecular chains can be advantageously investigated by Raman spectroscopy.

## Data Availability

Data available from the author.
